# Are we happy with the impact factor?

**DOI:** 10.3402/ejpt.v5.26084

**Published:** 2014-10-23

**Authors:** Miranda Olff

## Impact or impact factor

Which journal should I choose for the publication of my research? Which indicators of quality are important? How do I best reach a wide and international audience? Which journal within my field has most impact or the highest Impact Factor? In the end, what I'm looking for is a high-quality venue that at the same time allows my paper to be read, used, and cited by as many readers as possible.

Although many researchers see the publication of their works in Open Access journals as the most efficient way to increase impact simply because they are freely accessible, many academics are at the same time expected, and sometimes even required, to publish in journals that are covered by Web of Science (WoS) and have an impact factor.

Thus, when speaking about a journal's impact, most often we mean the impact factor as it is calculated by the Institute of Scientific Information (ISI, Thomson Reuters). It refers to the frequency with which a journal's articles are cited in other scientific publications with an impact factor over a period of time. However, the impact factor is now perceived as more than just a measure of citations; it has become a metric used to evaluate the researcher himself/herself and even academic institutions. Often, other types of clinical, societal, or other impacts are ignored in this context, which has been heavily criticized over the years (e.g. Williams, 2007).

## *EJPT* in WoS

In December 2013, the *European Journal of Psychotraumatology* (*EJPT*) was accepted in WoS, and the first impact factor (for 2013) was expected in June 2014. Belonging to the 10% of journals accepted into WoS, *EJPT* had proved that it had high “scientific quality.” However, when the Journal Citations Report came out this summer, *EJPT* was missing. After inquiry at Thomson Reuters, we were told that the volume of *EJPT* papers was very high and that they had not managed to create the citation reports in time. In March 2014, Thomson Reuters should have indexed all papers published in *EJPT* from the start of the journal in 2010 through 2013, but they were not and therefore *EJPT* will have to wait until June 2015 to get its first official impact factor.

Luckily the impact factor is quite easy to calculate. In any given year, it is calculated by counting all citations during one year to a journal's content published in the preceding 2 years, divided by the number of substantive, scholarly items published in those same 2 years (Garfield, 2006):

$$\text{IF}=\frac{\text{Number of citations in a given year}}{\text{Number of source articles in the previous 2 years}}$$

The items counted in the denominator as citable items are usually research articles, reviews, but not editorials or letters to the editor (except when they function as “articles”). There is ongoing debate on what exactly should go into the formula (McVeigh & Mann, 2009). Both in the numerator and denominator, only journals that are included in WoS are considered—all other sources are disregarded.

For *EJPT*, the 2013 unofficial impact calculation is slightly above 2 (see www.ejpt.net). Considering that the journal has only existed for 3½ years, we are quite pleased with that score.

## Transitions in science and new metrics

Open Access is politically supported in many countries as well as on a European level (see **here** for EU Open Access policy initiatives). The European Commission requires that by 2016 at least half of the scientific publications, the research of which it funds, must be published under an Open Access model—freely available to anybody, anywhere. This is also what Sander Dekker, the Dutch State Secretary for the Ministry of Education, Culture and Science, **announced on June 7**, 2014 (in Dutch) “Science is in need of fundamental reform.” It is hard not to agree. **Science in Transition** is a strong movement in the Netherlands with broad political support, challenging many aspects of science. “Science has become a self-referential system where quality is measured mostly in bibliometric parameters and where societal relevance is undervalued” say the founders of the movement (Dijstelbloem, Huisman, Miedema, & Mijnhardt, 2013).

Times are changing. Although ISI/Thomson Reuters have succeeded in setting a standard, it lacks the kind of refinement in a new globalized world where many other ways of measuring impact are possible and should also be accounted for, and for which authors should be credited. Rather than measuring the *journal's* impact, which may unfairly depend on one or a couple of much-cited articles and the rest with no citations at all, identifying an *author*'*s* impact (article level metrics, or altmetrics) is now becoming a more and more important alternative to the impact factor (see also Olff, 2013). Altmetrics cover not just citation counts but also other aspects of the impact of an article such as how many data and knowledge bases refer to it, article views, full-text downloads, Facebook likes, or mentions in social media and news media. **Click on** this article from the 2013 volume of *EJPT*, for example, and see what it may look like.

As regards the *EJPT*, we certainly cannot afford to undervalue its societal relevance, and—at the same time—we will have an impact factor next year!

*Miranda Olff* Editor-in-Chief
